# Detection of *Acinetobacter Baumannii* and *Staphylococcus Capitis *in Bile from Two Patients with Chronic Xanthogranulomatous Cholecystitis: The Impact of Metagenomic Analysis

**DOI:** 10.14789/jmj.JMJ21-0046-CR

**Published:** 2022-06-20

**Authors:** MASAAKI MINAGAWA, TERUO KIRIKAE, MARI TOHYA, YUKI FUKUMURA, AYAKO NAKAMURA, DAISUKE MOTOOKA, SHOTA NAKAMURA, TETSUYA IIDA, AKIO SAIURA, SHIN WATANABE

**Affiliations:** 1 Department of Hepatobiliary-Pancreatic Surgery, Juntendo University, Graduate School of Medicine, Tokyo, Japan; 1 Department of Hepatobiliary-Pancreatic Surgery, Juntendo University, Graduate School of Medicine, Tokyo, Japan; 2 Department of Microbiome Research, Juntendo University Graduate School of Medicine, Tokyo, Japan; 2 Department of Microbiome Research, Juntendo University Graduate School of Medicine, Tokyo, Japan; 3 Department of Microbiology, Juntendo University, Graduate School of Medicine, Tokyo, Japan; 3 Department of Microbiology, Juntendo University, Graduate School of Medicine, Tokyo, Japan; 4 Department of Human Pathology, Juntendo University, Graduate School of Medicine, Tokyo, Japan; 4 Department of Human Pathology, Juntendo University, Graduate School of Medicine, Tokyo, Japan; 5 Department of Clinical Laboratory, Juntendo University, Graduate School of Medicine, Tokyo, Japan; 5 Department of Clinical Laboratory, Juntendo University, Graduate School of Medicine, Tokyo, Japan; 6 Department of Infection Metagenomics, Genome Information Research Center, Research Institute for Microbial Diseases, Osaka University, Suita, Japan; 6 Department of Infection Metagenomics, Genome Information Research Center, Research Institute for Microbial Diseases, Osaka University, Suita, Japan

**Keywords:** xanthogranulomatous cholecystitis, *Acinetobacter baumannii*, *Staphylococcus capitis*, metagenomic analysis

## Abstract

**Background:**

*Escherichia coli* is thought to cause xanthogranulomatous cholecystitis (XGC). However, it is unclear whether other pathogens are associated with the cause and progression of XGC.

**Case presentation:**

Patient 1 was a 55-year-old man with a previous surgical history of right lung cancer. He presented with abdominal pain and was diagnosed with acute cholecystitis. He underwent endoscopic nasogallbladder drainage (ENGBD), antimicrobial therapy, and endoscopic sphincterotomy (EST). He underwent cholecystectomy on day 59. The patient was pathologically diagnosed with chronic phase XGC. *Acinetobacter baumannii* was isolated from the bile sample during the operation. Patient 2 was a 58-year-old man with no previous medical history. He presented with abdominal pain and was diagnosed with acute cholecystitis. He underwent endoscopic retrograde biliary drainage (ERGBD) and antimicrobial therapy. His symptoms improved, but acute cholecystitis became exacerbated on day 53. The patient was treated with antimicrobial therapy. He underwent cholecystectomy on day 88. The patient was pathologically diagnosed with focal acute inflammatory phase XGC. *Staphylococcus capitis* was isolated from the bile during the operation. This study describes two patients with XGC, one infected with *A. baumannii* and the other with *S. capitis*, in their gallbladders, which was identified by bacterial culture. Metagenomic analysis revealed that the genera *Acinetobacter* and *Staphylococcus* predominated and that other genera, including *Delftia* and *Anaerobacillus*, were also present, suggesting that these bacteria play a significant role in the pathological changes associated with XGC

**Conclusions:**

This is the first report of *A. baumannii* and *S. capitis* infections in patients with XGC.

## Introduction

*Xanthogranulomatous cholecystitis* (XGC) is characterized histologically by the accumulation of numerous foamy macrophages in the gallbladder, resulting in thickening of the gallbladder wall^1)^. Moreover, XGC is frequently diagnosed as gallbladder carcinoma. *Escherichia coli* antigens have been detected immunohistologically in XGC lesions^2)^, and *E. coli* was reported to be involved in the pathogenesis of XGC, with scavenger receptor class A and CXCL16-CXCR interactions^3)^. These results suggested that *E. coli* infections of the gallbladder play an important role in the onset and/or early stages of XGC. However, it is unclear whether other pathogens are associated with the cause and progression of XGC.

This study describes the isolation of two other species of bacteria, *Acinetobacter baumannii* and *Staphylococcus capitis*, from the bile samples of two patients with XGC. Metagenomic analysis of the microbiota in bile samples confirmed that these pathogens, as well as other microorganisms, were present in bile.

## Case report

Two patients (P1 and P2) underwent cholecystectomy for suspected chronic cholecystitis in July 2018 at the Department of Hepato-biliary Pancreatic Surgery, Juntendo University Hospital. Written informed consent was obtained from these patients before surgery. This study was conducted according to the principles of the Declaration of Helsinki, and approved by the Juntendo University ethics committee (JHS 18-060 Juntendo University Hospital Independent Ethics Committee). The clinical characteristics of these two patients are summarized in Table 1. Chronic cholecystitis in these patients was diagnosed as XGC by histopathological examination of the gallbladder samples.

**Table 1:** Clinical presentations and results of bacterial culture

Patient	P1	P2
CHARACTERISTICS OF PATIENTS		
Age Sex	55M	58M
Past medical history	Right lung cancer Af	NA
Cause of acute cholecystitis	Cholesterol stones	Cholesterol stones
LABORATORY DATA		
White blood cell count(cells/mm3)	15500	9600
C-reactive protein (mg/dL)	21	26
CEA(ng/ml) (< 5.0) / CA19-9(U/ml) (< 37.0)	3.1 / 11	1.9 / 13
PREOPERATIVE CHARACTERISTICS		
Time from onset to operation (days)	59	88
Drainage	ENGBD EST	ERGBD
Antimicrobial agents	CMZ MEPM	CMZ PIPC/TAZ CTRX LVFX
Discharge (Postoperative day)	7	5
TG 18 SEVERITY CLASSIFICATION		
Mild/Moderate/Severe	Moderate	Moderate
Blood culture	Negative	Negative
Bacterial profile of bile culture	Acinetobacter baumanii	Staphylococcus capitis
OPERATION		
Operation	Laparotomy cholecystectomy	Laparoscopic cholecystectomy

※NA, not applicable; Af, Atrial fibrillation; ERGBD, Endoscopic retrograde gallbladder drainage; ENGBD, Endoscopic nasogallbladder drainage; EST, Endoscopic sphincterotomy; CMZ, cefmetazole; PIPC/TAZ, Piperacillin/Tazobactam; CTRX, Ceftriaxone; LVFX, Levofloxacin; MEPM, Meropenem

**Figure 1:**
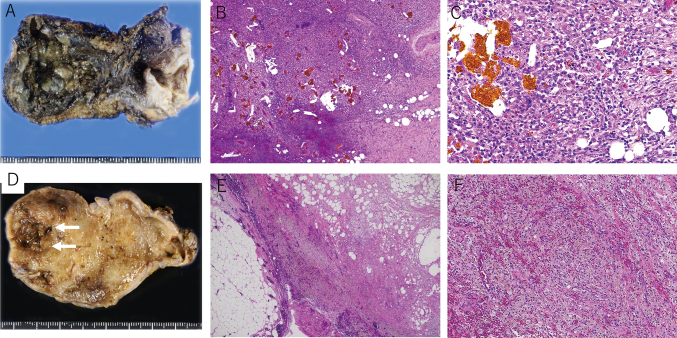
Pathological view of the 2 cases of gallbladder with xanthogranulomatous cholecystitis 1A-C: Patient 1. A: Grossly, a thickened black gallbladder is seen. B and C: Microscopically, diffuse infiltration of foam cells and lymphocytes are observed along with bile and cholesterol deposits. 1D-F. Patient 2. D. Grossly, a thickened gallbladder with coarse mucosa at the fundus (Arrows) is seen. E and F: Microscopically, diffuse infiltration of foam cells with bile pigment is seen.

**Figure 2:**
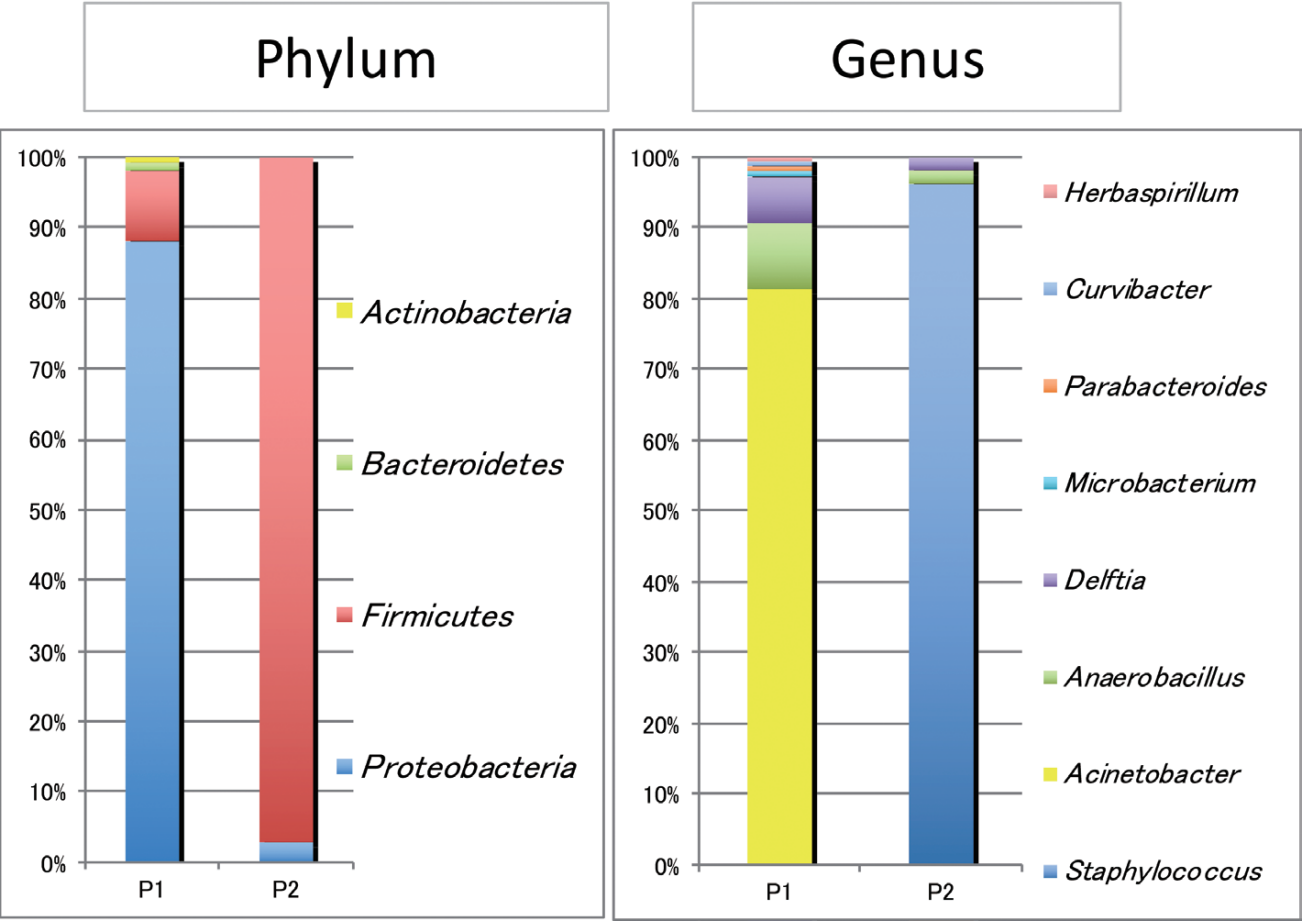
Relative abundance of major bacteria in bile

Patient 1 was a 55-year-old Japanese man with a previous surgical history of right lung cancer seven years earlier, and was admitted to an intensive care unit (ICU) with a mechanical ventilator. He presented with abdominal pain and was diagnosed with acute cholecystitis. He underwent endoscopic nasogallbladder drainage (ENGBD) on the first day and was treated with 3 g/day cefmetazole (CMZ) for three days. The bile sample obtained during ENGBD was negative for bacterial culture, but his AST/ALT was elevated. Endoscopic sphincterotomy (EST) was performed on the third day, and he was treated with 1.5 g/day meropenem (MEPM) for three days, followed by 3 g/day CMZ for three days. His symptoms disappeared and the patient was not administered any antimicrobial agents after day 10. He underwent cholecystectomy on day 59. Gross examination of the resected gallbladder showed the hemorrhagic mucosa with marked wall thickness (Figure 1A). Microscopic examination showed diffuse infiltration of foam cells along with multinucleated giant cells, lymphocytes, and cholesterol deposit (Figure 1B, C). No bacterial colonies or neutrophilic reactions were evident histologically. The patient was pathologically diagnosed with chronic phase XGC. *A. baumannii* was isolated from the bile sample obtained from Patient 1 during the operation. This isolate was susceptible to all drugs tested (Table 2). Metagenomic analysis of the bile sample from Patient 1 showed bacterial DNA derived from seven genera, with the genus *Acinetobacter* being predominant. Filtering of the data sets to include OTUs present in > 0.5% of the samples revealed bacterial DNA from four phyla, *Actinobacteria* (0.8%), *Bacteroidetes* (1%), *Firmicutes* (10%) and *Proteobacteria* (88%) (Figure 2).

**Table 2:**
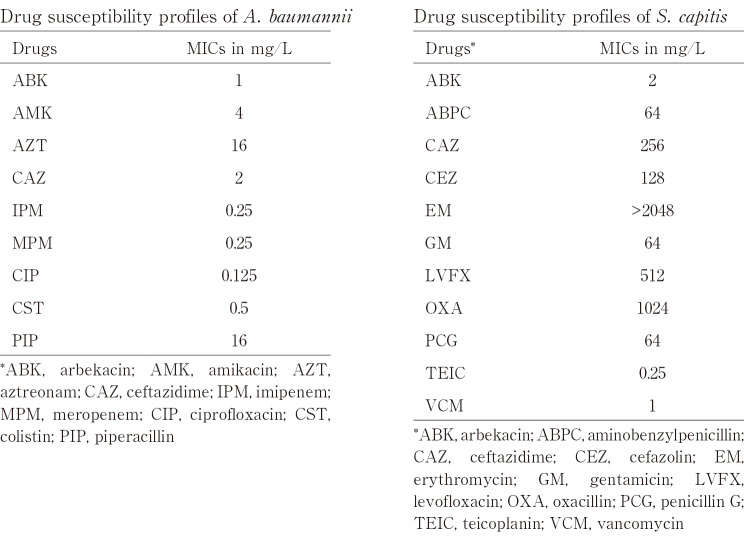
MIC and susceptibility of isolated bacteria to antibiotics

Patient 2 was a 58-year-old Japanese man with no previous medical history. He presented with abdominal pain and was diagnosed with acute cholecystitis. He underwent endoscopic retrograde biliary drainage (ERGBD) on the first day, and was treated with 6 g/day CMZ for two days, followed by 18 g/day piperacillin-tazobactam (PIPC/TAZ) for five days. His symptoms improved, but acute cholecystitis became exacerbated on day 53. The patient was treated with 1 g/day ceftriaxone (CTRX) for one day, followed by 0.5 g/day levofloxacin (LVFX) for seven days. His symptoms disappeared and the patient was not administered any antimicrobial agents after day 60. We adjusted the waiting period for a month to improve the inflammation, and he underwent cholecystectomy on day 88. Gross examination of the resected gallbladder showed that rough and coarse mucosa at the fundus with marked wall thickness and microscopic examination showed infiltration of bile containing foam cells along with lymphocytes (Figure 1D-F). Gram staining showed no evident bacterial colonies, although some neutrophilic reactions were observed, histologically. The patient was pathologically diagnosed with focal acute inflammatory phase XGC. *S. capitis* was isolated from the bile sample obtained from Patient 2 during the operation, with this isolate being resistant to ABPC, CAZ, EM, GM, LVFX, OXA, and PCG, but susceptible to ABK, TEIC, and VCM (Table 2). Metagenomic analysis of the bile sample from Patient 2 showed bacterial DNA derived from four genera, with the genus *Staphylococcus* being predominant. Filtering of the data sets revealed bacterial DNA from two phyla, *Firmicutes* (97%) and *Proteobacteria* (3%) (Figure 2).

### Laboratory procedure

During cholecystectomy, bile samples were obtained under sterile conditions from the fundus of the gallbladder using an 18G needle and 20 ml syringe. A portion of each was immediately frozen at -80℃ for metagenomic analysis, and the remaining bile samples were sent to the bacteriologic laboratory for culture. Samples were inoculated onto 5% sheep blood trypticase soy agar (Nissui Pharmaceutical, Japan), Drigalski agar (Nissui Pharmaceutical) and Anaero Columbia agar RS (Becton Dickinson and Company, Japan), and incubated in an atmosphere containing 10% CO_2_ at 35℃ for 5 days. The isolates were identified using the MALDI biotyper (Bruker Co., Ltd., Japan) over a score of 1.700. The minimum inhibitory concentrations (MICs) of antimicrobial agents were determined using the microdilution method, according to the guidelines of the Clinical & Laboratory Standards Institute (CLSI, M100-S25). The antimicrobial agents tested included amikacin (AMK), aminobenzylpenicillin (ABPC), arbekacin (ABK), aztreonam (AZT), cefazolin (CEZ), ceftazidime (CAZ), ciprofloxacin (CIP), colistin (CST), erythromycin (EM), gentamicin (GM), imipenem (IPM), levofloxacin (LVFX), meropenem (MEPM), oxacillin (OXA), penicillin (PCG), piperacillin (PIP), teicoplanin (TEIC) and vancomycin (VCM). Genomic DNA of bacterial isolates was extracted using DNeasy Blood & Tissue kits (QIAGEN, Tokyo, Japan). Genomic libraries were prepared using Nextera XT DNA kits (Illumina, San Diego, CA). Paired-end sequencing was performed using MiSeq Reagent Kit v3 (600-cycles). Quality trimming, filtering and assembly of the obtained sequence reads were performed using CLC Genomics Workbench v11 (QIAGEN, Hilden, Germany). The assembled genome sequence data were searched for genes associated with drug resistance, using the ABRicate program (https://github.com/tseemann/abricate) and data from the National Center for Biotechnology Information (NCBI), the Comprehensive Antibiotic Resistance Database (CARD), and the ResFinder databases.

DNA was extracted from bile samples using DNeasy PowerSoil Kits (QIAGEN). Each library was prepared in accordance with “Illumina 16S Metagenomic Sequencing Library Preparation Guide” with a primer set (27Fmod: 5ʼ- AGR GTT TGA TCM TGG CTC AG -3ʼ and 338R: 5ʼ- TGC CTC CCG TAG GAG T -3ʼ) targeting the V1–V2 region of the 16S rRNA gene. The 251 bp paired end sequencing of the amplicon was performed on a MiSeq. The obtained paired end sequences were merged using PEAR (http://sco.h-its.org/exelixis/web/software/pear/). Subsequently, 30,000 reads per sample were randomly sampled using seqtk (https://github.com/lh3/seqtk) for taxonomic assignment. These sampled sequences were clustered into operational taxonomic units (OTU) defined as 97% similarity using UCLUST version 1.2.22q. Representative sequences for each OTU were classified taxonomically using RDP Classifier version 2.2 with the Greengenes database (gg_13_8).

The isolate harbored 10 genes associated with drug resistance, including *AAC*(*6’*)-*Ie-APH*(*2”*)- *Ia*, *ant(9)-Ia*, *blaI*, *blaR1*, *blaZ*, *ermA*, *mecA*, *mecI*, *mecR1*, *and tetM*.

## Discussion

Because *E. coli* is the organism most frequently isolated from bile samples of patients with cholecystitis, it has been regarded as a cause of this condition. Other microorganisms isolated from the bile samples of patients with cholecystitis include *Enterobacter*, *Enterococcus*, *Klebsiella*, *Streptococcus*, and *Pseudomonas* spp.^4)^. These pathogens are thought to enter the gallbladder from the duodenum in a retrograde manner, although there is other possibility that they enter the gall bladder via the portal vein through the hepatic sinusoids and space of Disse^5)^. Gallbladder stones play an important role in the pathological conditions observed in patients with cholecystitis.

Pathologically, XGC is characterized by thickening of the gallbladder wall, mimicking advanced gallbladder carcinoma^1)^. Although these pathological changes are thought to be due to intense acute or chronic inflammation, the pathogenesis of XGC remains unclear. XGC is often associated with gallstones. Gallstones cause ulceration of the gallbladder mucosa, rupture of Rokintansky-Aschoff sinuses, and eventually xanthogranulomatous changes^6)^.

Next-generation DNA sequencing has enabled analysis of the microbiota in the biliary tracts of patients with various diseases, including bacterial infection associated with acute cholecystitis^7)^, chronic cholecystitis^8)^, and XGC. This study found that *A. baumannii* and *S. capitis* were present in the bile samples of two patients with XGC, suggesting that bacteria other than *E. coli* contribute to pathological changes in XGC. To our knowledge, no previous study has reported that other species of bacteria, including *A. baumannii* and *S. capitis*, contribute to XGC. Two types of nephritis pathologically similar to XGC, xanthogranulomatous pyelonephritis and urinary malakoplakia, are caused by *Enterobacteriaceae*, including *E. coli* ^9, 10)^. Histologic examination of gallbladder samples showed localized accumulation of abundant foamy macrophages^1, 9, 10)^, with these pathological changes induced by *E. coli* infection^2, 3)^.

*A. baumannii* is a Gram-negative, opportunistic pathogen that can survive on solid and dry surfaces for up to 5 months. *A. baumannii* can grow over wide ranges of temperature and pH and forms biofilms on abiotic substrates^11)^. *A. baumannii* may have been present in bile when Patient 1 was admitted to the ICU.

*S. capitis* is a coagulase-negative staphylococcus with documented potential for both human diseases and nosocomial spread^12)^. *S. capitis*, which causes prosthetic valve endocarditis and joint infection^12)^, is mainly distributed on the head (primary ears and forehead), arms and occasionally the legs^13)^. The *S. capitis* isolate obtained from the Patient 2 was multidrug-resistant, suggesting that this patient must have been infected with this pathogen during in-hospital treatment with antibiotics. In addition, the procedures used during ERCP may have been responsible for the entry of this pathogen into the biliary tract.

To our knowledge, this is the first report of *A. baumannii* and *S. capitis* infections in patients with XGC. *Delftia* and *Anaerobacillus*, as well as other several genera, may play a role in the histopathological changes involved in progression from chronic cholecystitis to XGC. However, it is unclear whether these pathogens directly contribute to the pathological change of XGC. Further studies are needed to analyze the bacteriology and metagenome involved in gallbladder diseases. Metagenomic analysis is a useful tool for detection of pathogens in chronic cholecystitis such as XGC.

## Declarations

### Ethics approval and consent to participate

This study was conducted according to the principles of the Declaration of Helsinki, and approved by the Juntendo University ethics committee (JHS 18-060 Juntendo University Hospital Independent Ethics Committee).

## Consent for publication

Written informed consent was obtained from these patients before surgery for publication of this case report.

## Availability of data and materials

All sequence data of the 16S rRNA sequences for metagenome analysis and two isolates in this study were deposited in the DDBJ/GenBank/EMBL database under accession number DRA007947, DRA007974 and DRA007975, respectively.

## Funding

This work was supported by Asahi Group Holdings, Ltd. Japan. The funders had no role in the study design, data collection and analysis, decision to publish, and preparation of the manuscript.

## Author contributions

MM and AS treated the patient. MT, TK, and AN assessed the bacteriological analysis. DM, SN, and TI assessed the microbial analysis. YF performed pathological evaluation. MM, SW, and TK drafted the manuscript and responsible for study design. All authors read and approved the final manuscript.

## Conflicts of interest statement

The authors declare that they have no competing interests.
